# MicroRNA‐194 is a Marker for Good Prognosis in Clear Cell Renal Cell Carcinoma

**DOI:** 10.1002/cam4.631

**Published:** 2016-02-10

**Authors:** Roy Nofech‐Mozes, Heba W. Z. Khella, Andreas Scorilas, Leza Youssef, Sergey N. Krylov, Evi Lianidou, Konstantinos G. Sidiropoulos, Manal Gabril, Andrew Evans, George M Yousef

**Affiliations:** ^1^ Department of Laboratory Medicine and the Keenan Research Centre for Biomedical Science at the Li KaShing Knowledge InstituteSt. Michael's HospitalTorontoM5B 1W8Canada; ^2^ Department of Laboratory Medicine and PathobiologyUniversity of TorontoTorontoONM5S 1A8Canada; ^3^ Departments of Biochemistry and Molecular BiologyUniversity of Athens15701AthensGreece; ^4^ Departments of Chemistry and Centre for Research on Biomolecular InteractionsYork UniversityTorontoOntarioM3J 1P3Canada; ^5^ Department of ChemistryUniversity of Athens15771AthensGreece; ^6^ Department of PathologyLondon Health Sciences Center and Western UniversityLondonN6A 5W9Canada

**Keywords:** Kidney cancer, metastasis, miR‐194, miRNA, personalized medicine, prognosis, prognostic marker, renal cell carcinoma, survival, tumor markers

## Abstract

Clear cell renal cell carcinoma (ccRCC) is the most prevalent adult kidney cancer. Prognostic markers are needed to guide patient management toward aggressive versus more conservative approaches, especially for small tumors ≤4 cm. miR‐194 was reported to be downregulated in several cancers and is involved in epithelial to mesenchymal transition. We evaluated miR‐194 as a prognostic marker in ccRCC. In a cohort of 234 patients with primary ccRCC, we correlated miR‐194 expression level with multiple clinicopathological features including disease‐free and overall survival, tumor size, clinical stage, and histological grade. Our results shows a stepwise decrease in miR‐194 expression from normal kidney to primary ccRCC (*P *= 0.0032) and a subsequent decrease from primary to metastatic lesions. Additionally, patients with higher miR‐194 expression has significantly longer disease‐free survival (*P* = 0.041) and overall survival (*P* = 0.031) compared to those with lower expression. In multivariate analysis, miR‐194‐positive tumors retain significance in disease‐free survival and overall survival, suggesting miR‐194 is an independent marker for good prognosis in ccRCC. Moreover, miR‐194 is a marker for good prognosis for patients with small renal masses (*P* = 0.014). These findings were validated on an independent data set from The Cancer Genome Atlas. We also compared miR‐194 expression between RCC subtypes. ccRCC had the highest levels, whereas chromophobe RCC and oncocytoma had comparable lower levels. Target prediction coupled with pathway analysis show that miR‐194 is predicted to target key molecules and pathways involved in RCC progression. miR‐194 represents a prognostic biomarker in ccRCC.

## Introduction

Renal Cell Carcinoma (RCC) is a cancer of the renal parenchyma and the most common type of adult kidney cancer, making up 90% of renal malignancies 1. The incidence of RCC is increasing over recent decades, 2 which may be related to the advanced diagnostic techniques for early detection of small renal masses 3, 4 and increased rates of risk factors such as obesity and hypertension 5. Disease stage is currently the most important factor that correlates with disease survival. Patients with metastatic disease have a median survival of 19 months 6. RCC is histologically divided to a number of subtypes, the most prevalent of which is the clear cell (ccRCC) which is associated with *VHL* gene inactivation 7.

Early‐stage ccRCC is clinically silent and therefore detection is often incidental by imaging, especially for patients with small renal masses (pT1a, ≤4 cm). Patients with larger tumors are diagnosed during various stages of their disease, including 20–30% of ccRCC patients already presenting with advanced disease or metastasis 8. The current therapeutic approach for localized ccRCC is partial or radical nephrectomy, while in patients with advanced RCC, cytoreductive nephrectomy and systemic‐targeted therapy were shown to increase the overall survival 6.

In patients with small renal masses and localized ccRCC, it is difficult to predict recurrence or progression to metastasis. Several prognostic scoring algorithms incorporate tumor size, stage, grade, histologic necrosis, and regional lymph node 9. Memorial Sloan Kettering Cancer Center developed a prognostic algorithm for metastatic RCC patients based on clinical parameters that was validated and modified for patients under targeted therapies 10. There is still an urgent need for prognostic markers that could predict disease aggressiveness and survival at an early stage based on molecular parameters independent of tumor morphology. These can greatly impact patient management 11.

In kidney cancer, as is the case in other cancers, there is a trend for less aggressive therapy (active surveillance) for nonprogressive small renal masses (pT1a ≤ 4 cm). Unfortunately, all patients are currently treated by nephrectomy due to the lack of prognostic markers that can distinguish between progressive and nonprogressive tumors. Moreover, renal RCC are aggressive tumors with ~35% chance of tumor spread and metastasis. This is the leading cause of death in kidney cancer. There is an urgent need to predict tumor behavior at the time of resection, so that patients with aggressive disease can be candidates for closer follow‐up ± adjuvant therapy, while those with less aggressive form of the disease can enjoy less frequent follow‐up. This marker can be incorporated to other clinicopathologic parameters to improve prognostic perdition. Recently, a number of molecular markers have been identified 12, 13.

A class of molecules which have garnered serious consideration as biomarkers are microRNAs (miRNAs). miRNAs are short noncoding RNA molecules that are posttranscriptional repressors of protein‐coding genes. Through a specific binding to the 3′‐UTR, miRNAs decrease gene expression by blocking translation or degrading the mRNA. miRNAs are deregulated in a many cancers including ccRCC 14. In addition to their functional role to promote or inhibit ontogenesis, miRNAs have a potential to be promising prognostic biomarkers. miR‐194 has been identified to play a role in several cancers, including hepatic, gastric, and colorectal cancers as well as ccRCC 15, 16, 17, 18. It has been described as a tumor suppressor miRNA that was shown to be involved in epithelial to mesenchymal transition (EMT) and suppression of metastasis 17.

We analyzed miR‐194 expression in primary ccRCC and examined its potential utility as a prognostic marker. We validated our results using TCGA (The Cancer Genome Atlas database). We finally explored the potential involvement of miR‐194 in ccRCC pathogenesis by in‐silico analysis.

## Materials and Methods

### Specimens collection

We analyzed a total of 234 ccRCC primary pretreatment formalin‐fixed paraffin‐embedded tissues from the archives of the department of pathology at St. Michael's Hospital, Canada from 2001–2009. Mean disease‐free survival (DFS) was 48.6 ± 2.19 months (1.0–120.0 months) and mean OS was 53.9 ± 2.12 months (1.0–131.0 months). Diagnoses were confirmed by a pathologist. Tissues were taken from areas with no hemorrhage or necrosis, and multiple sections were submitted from the same tumor to compensate for tumor heterogeneity. Tumor classification and staging were established according to the 2002 TMN System and the 2004 WHO classification. All procedures were carried out according to the Research Ethics Board approval from St. Michael's Hospital, Toronto, Canada. Also, RNA was extracted from 23 pairs of normal/cancer fresh tissues from the same patient for comparing miR‐194 expression between normal and cancerous tissues. The normal kidney tissues were taken from the kidney cortex of the same patient away from the tumor. We also compared the expression of miR‐194 in oncocytoma and different RCC subtypes using fresh tissues obtained from 20 samples for each group. Fresh specimens were collected immediately after resection, snap frozen in liquid nitrogen, and stored at −80°C until total RNA extraction.

### Total RNA extraction

Pure tumor areas were obtained by laser capture microdissection. Total RNA was extracted using miRNeasy (Siegen, Mississauga, Canada) according to the manufacture's protocol, as described in our recent publication 19. Total RNA concentrations were determined spectrophotometrically (NanoDrop 1000 Spectrophotometer, NanoDrop Technologies Inc., Wilmington, Delaware). Samples optimal for analysis were stored at −80°C.

### Quantitative real‐time RT‐PCR (qRT‐PCR)

Quantitative real‐time PCR (qRT‐PCR) was used to measure miRNA expression with TaqMan MicroRNA Assays (Applied Biosystems, Foster City, CA, USA) as described in our recent publication 19. MiR‐194‐specific reverse transcription was performed with 5 ng total RNA using the TaqMan^®^ MicroRNA Reverse Transcription Kit (Applied Biosystems) as recommended by the manufacturer. qRT‐PCR was performed using the TaqMan microRNA Assay^®^ Kit on the Step One^™^ Plus Real‐Time PCR System (Applied Biosystems). Thermal cycling conditions were according to the manufacturer's fast protocol and all reactions were performed in triplicate. Gene expression analysis was performed using the comparative C_T_ (2^−ΔΔC^_T_) method in order to calculate the relative quantification (RQ units) units of *miR‐194* in kidney tumors.

The comparative C_T_ method 2^−ΔΔCΤ^was used for performing relative quantification analysis. miR‐194 expression levels were normalized to the geometrical mean of two housekeeping genes RNU44 and RNU48.

### Statistical analysis

Owing to the non‐Gaussian distribution of miR194 in the patient cohort, Mann–Whitney *U* Test was run in order to analyze the association of miR194 expression levels, a continuous variable, with nominal parameters such as tissue status (primary or metastatic), and patients' sex (male or female). In case of ordinal variables, such as TNM stage (I/II/III/IV), their relation with miR194 expression levels (continuous variable) was estimated using Jonckheere–Terpstra Test. The Jonckheere trend test (Jonckheere–Terpstra test) is a test for an ordered alternative hypothesis within an independent samples design. The Jonckheere–Terpstra test is similar to the Kruskal–Wallis H test, but with more statistical power.

In order to determine the optimal cut‐off point for categorization of patients into miR‐194‐positive and miR‐194‐negative as there are no established cut‐off points, we used the X‐tile software (New Haven, CT), an algorithm that facilitated the determination of an optimal cut‐off point by correcting for the use of minimum *P*‐value statistics algorithm 20. For miR‐194 expression conversion to a dichotomous variable, an optimal cutoff of 0.28 RQ Units (equal to the 20th percentile) was produced using X‐Tile algorithm.

Hence, miR‐194 expression was categorized to positive or negative and associations between miR‐194 status and clinicopathologial variables were determined using Fisher's Exact Test or Pearson Chi‐square test. Cox proportional hazard regression analysis was performed at both univariate and multivariate levels. The multivariate model was adjusted for patients' sex, tumor size, and tumor grade and *P* values were calculated using the test for trend approach. In parallel, Kaplan–Meier curves were constructed, so that the percentage probability of patients' DFS and OS to be calculated. Differences between these curves were evaluated by the log‐rank test and the level of significance was set at a probability value of less than 0.05 (*P* < 0.05).

### Clinical validation on The Cancer Genome Atlas Dataset

We compiled miR‐194 normalized RPKM values (level 3) and clinical variables associated with ccRCC patients from 481 patients of The Cancer Genome Atlas . (www.cancergenome.nih.gov 21. Clinical variables that were analyzed in relation to miR‐194 read counts included overall survival time, pathologic stage, and tumor size. Cut‐off points were determined and Kaplan–Meir curves were constructed using cutoff finder software (http://molpath.charite.de/cutoff/index.jsp).

### Bioinformatic analyses

#### Target prediction and pathway analysis

Target prediction was done using TargetScanHuman 6.2 (http://www.targetscan.org/) and miRecords software (http://c1.accurascience.com/miRecords/prediction_query.php). Only predictions by at least three programs were included in the analysis. We filtered the predicted gene targets list through extensive literature search and pathway analysis using DIANAmirPath (http://diana.imis.athenainnovation.gr/DianaTools/index.php?r=mirpath/index) and the Gene Functional Classification tool from DAVID Bioinformatics Database (http://david.abcc.ncifcrf.gov/gene2gene.jsp).

## Results

### A stepwise downregulation of miR‐194from normal to primary then metastatic ccRCC

We first compared miR‐194 expression between cancerous tissues and normal counterpart from the same patient. miR‐194 is significantly lower in the cancerous tissue compared to adjacent normal kidney tissue. Out of 23 cases examined, 19 cases (83%) showed a pairwise decrease in cancer compared to normal (*P* = 0.0032) **(**Table 1). We next compared miR‐194 expression between 234 primary and 12 metastatic ccRCC. Mean miR‐194 expression was higher in primary compared to metastatic tumors (2.67 ± 0.90 and 1.30 ± 0.37, respectively), although this was not statistically significant (Table S1**).**

**Table 1:** Pairwise miR‐194 expression in ccRCC and adjacent normal kidney tissues from the same patient (*n* = 23)

Pairwise comparison^a^, ^b^: number of cases with		Average expression signal	
cancer >normal	Normal > cancer	Normal	Cancer
19	4	5787.85	3201.26

a Average pairwise fold change = 0.553.

b Pairwise *P*–value = 0.0032.

### miR‐194 is a potential independent prognostic marker for ccRCC

We tested the association between miR‐194 expression and different clinicopathological characteristics in primary ccRCC. As a binary variable, there was no significant association between miR‐194 expression level and tumor size, stage, or age. Interestingly, lower expression was seen in grade IV tumors compared to grade II‐III **(**Table**** 2). Interestingly, expression levels were lower in grade I compared to grade II and III. This might be explained by the small number of cases that had grade I (*n* = 14), or it might be a reflection of heterogeneity in tumor grading among pathologists.

**Table 2:** Associations between miR‐194 status^a^ and clinicopathological variables in ccRCC

Variable	Total	No. of patients (%)		*P*‐value
		miR194‐negative	miR194‐positive	
Sex				
Male	152	26 (17.1)	126 (82.9)	0.227^b^
Female	82	20 (24.4)	62 (75.6)	
Age (Years)				
≤61	117	23 (19.7)	94 (80.3)	1.000^b^
>61	117	23 (19.7)	94 (80.3)	
Laterality				
Left	132	26 (19.7)	106 (80.3)	1.000^b^
Right	102	20 (19.6)	82 (80.4)	
Tumor size (cm)				
≤4.0	79	15 (19.0)	64 (81.0)	0.863^b^
>4.0	154	32 (20.8)	122 (79.2)	
ΤΝΜ stage				
I	88	14 (15.9)	74 (84.1)	0.343^c^
*ΙΙ*	19	4 (21.1)	15 (78.9)	
*ΙΙΙ*	24	7 (29.2)	17 (70.8)	
IV	32	9 (28.1)	23 (71.9)	
Tumor grade				
I	14	5 (35.7)	9 (64.3)	0.016^c^
II	96	17 (17.7)	79 (82.3)	
III	87	11 (12.6)	76 (87.4)	
IV	31	11 (35.5)	20 (64.5)	

Status is unknown.

a Cut‐off point: 0.28 RQ Units, equal to the 20th percentile.

b Calculated using Fisher's Exact test.

c Calculated using Pearson Chi‐square test.

Our univariate analysis showed that miR‐194‐positive patients have a statistically significant longer disease‐free survival (HR = 0.56, 95% CI = 0.31–0.99, *P* = 0.046). Furthermore, miR‐194‐positive patients have longer overall survival, although this did not reach statistical significance(HR = 0.44, 95% CI = 0.19–1.00, *P* = 0.05). After controlling for sex, tumor size, and grade in the multivariate analysis, miR‐194‐positive patients showed statistically significant association with longer disease‐free and overall survival compared to those who are miR‐194‐negative (HR = 0.52, 95% CI = 0.27–0.98, *P* = 0.043; and HR = 0.35, 95% CI = 0.14–0.88, *P* = 0.026, respectively) **(**Table**** 3 **)**. These results support miR‐194 as an independent marker for good prognosis in ccRCC.

**Table 3:** miR‐194 expression and patients' survival

Variable	Disease‐free survival			Overall survival		
	HR^a^	95% CI^a^	*P*‐value	HR^a^	95% CI^b^	*P*‐value
Univariate analysis						
miR‐194						
Negative	1.00			1.00		
Positive	0.56	0.31–0.99	0.046	0.44	0.19–1.00	0.05
Sex	0.63	0.37–1.09	0.10	0.35	0.16–0.79	0.011
Tumor Size	1.23	1.16–1.30	<0.001	1.26	1.17–1.36	<0.001
Tumor Grade (Ordinal)	3.24	2.32–4.50	<0.001	2.64	1.37–5.08	0.004
Multivariate analysis^c^						
miR‐194						
Negative	1.00			1.00		
Positive	0.52	0.27–0.98	0.043	0.35	0.14–0.88	0.026
Sex	0.83	0.45–1.54	0.55	0.24	0.07–0.84	0.026
Τumor Size	1.22	1.12–1.33	<0.001	1.30	1.15–1.47	<0.001
Tumor Grade (Ordinal)	2.41	1.58–3.65	<0.001	1.88	1.00–3.54	0.05

a Hazard ratio, estimated from Cox proportional hazard regression model.

b Confidence interval of the estimated HR.

c Multivariate models were adjusted for patients' sex, tumor size, and tumor grade.

**Figure 1:**
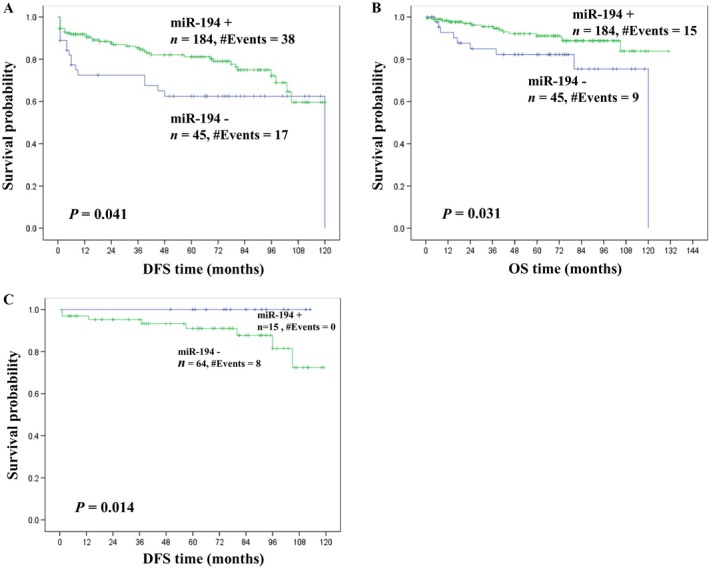
Kaplan–Meier survival curves showing significantly better prognosis in patients with tumors expressing higher levels of miR‐194, both in terms of disease‐free (DFS) (A) and overall survival (OS) (B). (C) Patients with small renal masses (pT1a; ≤4 cm) also show a disease‐free survival benefit when tumors expressed higher levels of miR‐194.

As shown in Figure**** 1, the Kaplan–Meier survival curves show that patients with higher miR‐194 expression had significantly longer disease‐free survival (*P = *0.041) and overall survival (*P* = 0.031) compared to those with lower expression levels. Further analyses were conducted for patient subgroups stratified based on tumor size and stage. Patients with small renal masses (pT1a; ≤4 cm) showed a disease‐free survival benefit when tumors expressed higher levels of miR‐194 (*P* = 0.014) (Fig.**** 1C).The same trend was noticed overall survival although it did not reach statistical significance (*P* = 0.29).

**Figure 2:**
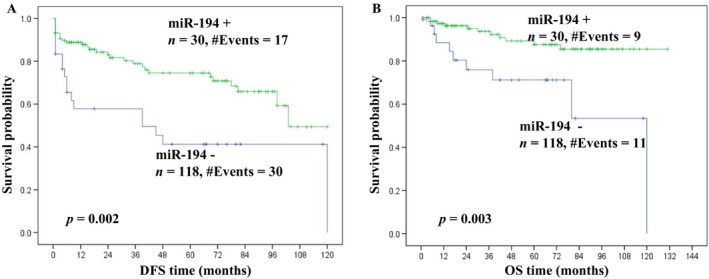
Kaplan–Meier curves show significantly better prognosis in patients with tumors >4 cm, expressing higher levels of miR‐194, both in terms of disease‐free survival (DFS) (A) and overall survival (OS) (B).

In the subgroup of patients with tumors >4 cm, those who were miR‐194 positive had significantly longer disease‐free and overall survival (*P* = 0.002 and *P* = 0.003, respectively) (Fig.**** 2 A–B).

### Validation of miR‐194 as a prognostic marker in ccRCC

**Figure 3:**
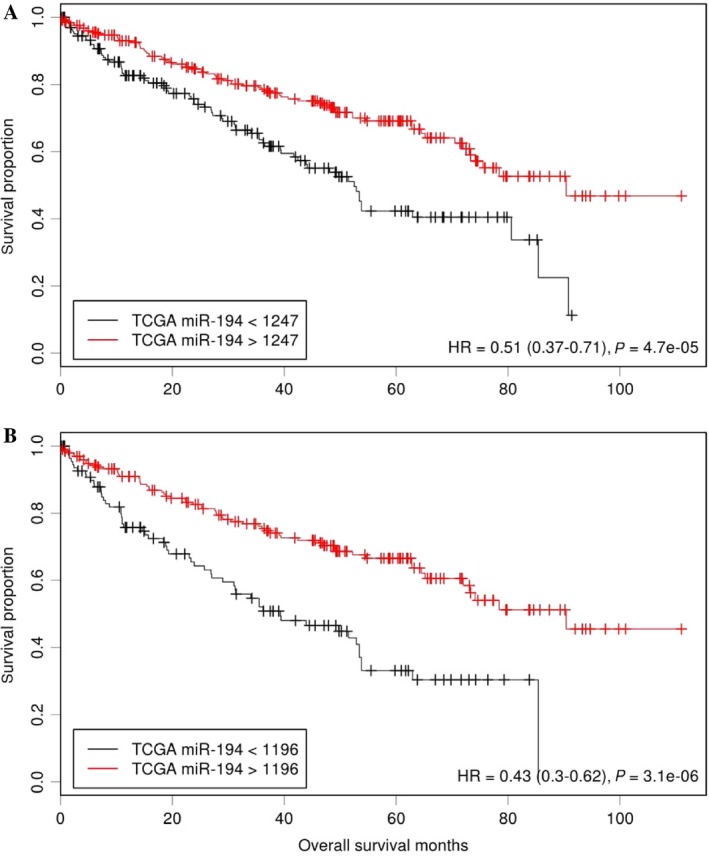
Kaplan–Meier curves showing the association of miR‐194 expression and survival in The Cancer Genome Atlas (TCGA) data set. (A) Patients with tumors expressing higher levels of miR‐194had significantly higher overall survival compared to those with lower miR‐194 expression. (B) In tumors larger than 4 cm, Kaplan–Meier curves indicate that patients with higher levels of miR‐194had significantly higher overall survival.

We validated our results in an independent dataset of 313 cases from TCGA. In this data set, we were able to verify our original findings regarding the relationship between miR‐194 and overall survival. Patients with miR‐194‐positive tumors had significantly higher overall survival (HR = 0.51, 95% CI = 0.37–0.71, *P = *4.7e–05) compared to patients with lower miR‐194 expression **(**Fig.**** 3A). Furthermore, we were able to validate that miR‐194 hold a robust prognostic significance for ccRCC tumors >4 cm in terms of overall survival (HR = 0.43, 95% CI = 0.3–0.62, *P = *3.1e–06) as shown in (Fig. 3B). miR‐194 expression was independent from pathologic stage and tumor size. Disease‐free survival information was not available for this dataset.

### miR‐194 expression in RCC subtypes

**Figure 4:**
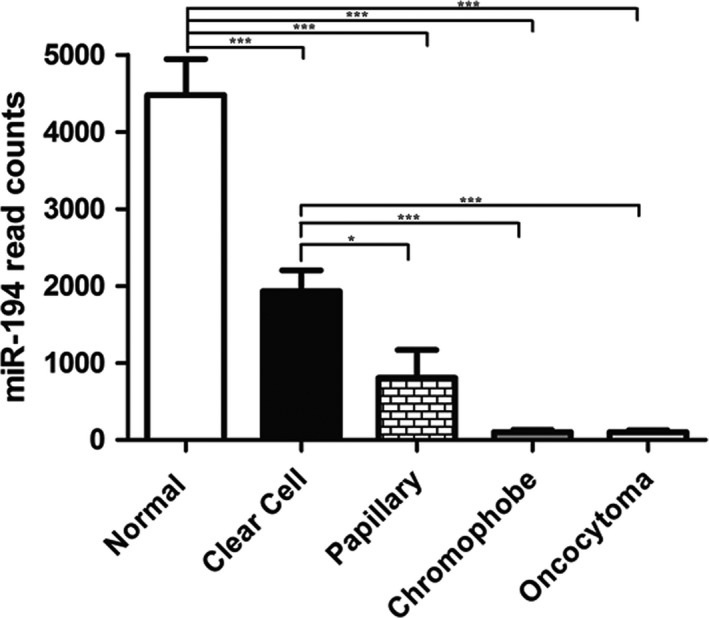
miR‐194 expression in common RCC subtypes and oncocytoma. The expression level of miR‐194 was significantly higher in the clear cell s compared to other subtypes. Both chromophobe RCC and oncocytoma had comparable much lower expression levels.

We compared miR‐194 expression between different RCC subtypes. The expression level of miR‐194 was significantly higher in the clear cell subtype in comparison to papillary or chromophobe RCC as well as oncocytomas **(**Fig. 4). Interestingly, miR‐194 expression levels were comparable in chromophobe RCC and oncocytoma, in keeping with recent literature suggesting that these two lesions represent two ends of the same spectrum.

### miR‐194 targets critical pathways and key molecules involved in tumor progression

In order to explore the potential involvement of miR‐194 in RCC aggressive behavior, we performed target prediction coupled with pathway analysis. Our results show that miR‐194 targets key molecules involved in tumor progression including *HIF1A, MDM2,PIK3R2, MAPK1, IGF1R,BCL2, ITGB1*, and *CRK*. Also, pathway analysis showed that miR‐194 targets critical pathways including the *HIF‐hypoxia pathway*,* VEGF, mTOR*,* Wnt, TGF‐beta*, and *MAPK* signaling pathways **(**Table S2**).**

## Discussion

Our results are in accordance with recent literature showing a gradual decrease in miR‐194 expression from normal tissue to primary to metastatic tumors 15, 18, 22, 23. Other miRNAs have shown this stepwise reduction in ccRCC 24. Downregulation can be attributed to multiple factors such as epigenetic changes, mutations, as well as alterations to transcription factors such as *p53*, which induces a miR‐194 25, 26, 27, 28. Alternatively, miR‐194 targets *MDM2*, which is a repressor of *p53* 29, 30. The autoregulatory loop of p53/MDM2 has been shown to be impaired by downregulated miR‐194 in other cancers 31.

Predicted targets of miR‐194 in RCC include *HIF1A, MAPK1, RAP1B, AKT2;* which are major components of the ccRCC pathogenesis as well as other oncogenic pathways 23. The deregulation of miR‐194 in metastasis is not surprising. Metastasis is often associated with epithelial to mesenchymal changes. miR‐194 contribute to two miRNA clusters (miR‐192‐miR‐194 and miR‐194‐miR‐215), which are documented to regulate EMT. These tumor suppressing miRNAs have been shown to limit cellular invasion and migration. Similarly, there is also evidence showing that miR‐194 suppresses metastasis in liver cancer 17. In gastric cancer cells, miR‐194 is shown to inhibit cell migration, invasion, as well as the EMT phenotype through targeting of the *FoxM1* 16, 32. Also, miR‐194 was shown to inhibit EMT in endometrial cancer cells 33. Our target prediction analysis showed that miR‐194 targets a number of pathways that can be related to EMT 34, 35. We have validated a number of miR‐194 target interaction in our previous work and demonstrated that overexpression of miR‐194 reduced cellular invasion and migration in renal cell carcinoma 15. Furthermore, miR‐194 target interaction was previously validated and the effect of miR‐194 overexpression on different cellular processes including cellular migration and invasion was previously demonstrated in different cancers 33, 34.

Our results show that miR‐194 is expression is differentially expressed between RCC subtypes. This is in keeping with recently published results 36. It has also been shown to be downregulated in nephroblastomas 22. The differential expression between the subtypes might be a reflection of distinct pathogenesis in each and might have therapeutic implications. There is a therapeutic potential for treatment using a miR‐194 mimics that can enhance the tumor suppressor function of miR‐194 including EMT suppression and consequently decrease metastatic potential.

Our results show miR‐194 can also be used to identify aggressive small renal masses (pT1a ≤ 4 cm) with worse prognosis and this can have a significant impact on treatment decision where more indolent tumors can be treated with more conservative approaches like active surveillance or local ablation, whereas surgical resection will be reserved for ccRCC small renal masses with predicted aggressive behavior. As a prognostic marker, miR‐194 expression can be also incorporated into prognostic algorithms to enhance their accuracy.

While the results are compelling, there is still a need for further validation in a larger prospective cohort of patients. Cutoffs for expression of miR‐194 were based upon this study population and technique used to evaluate expression.

In conclusion, miR‐194 is useful as a prognostic marker in ccRCC and can be used to compliment other biomarkers to predict disease relapse and overall survival. In patients with small renal masses, where treatment plans may vary, miR‐194 expression in the tumor is a useful piece of information in identifying an aggressive tumor with a high potential to relapse.

## Conflict of Interest

None declared.

## Supporting information


**Table S1.** Comparison of miR‐194 expression between primary and metastatic ccRCC.
**Table S2.** miR‐194 is predicted to target key molecules and pathways involved in RCC progression.
