# Supplementary material for: MicroRNA‐194 is a Marker for Good Prognosis in Clear Cell Renal Cell Carcinoma
Source: Cancer Med. 2016 Feb 10;5(4):656–64. doi: 10.1002/cam4.631 (PMC4831284; doi:10.1002/cam4.631)
Supplement: Supplementary file 1 — Table S1. Comparison of miR‐194 expression between primary and metastatic ccRCC. Table S2. miR‐194 is predicted to target key molecules and pathways involved in RCC progression. [file CAM4-5-656-s001.docx]

**Supplementary Table 1.**Comparison of miR-194 expression between primary and metastatic ccRCC.

|  |  |  | **Percentile** | | |
| --- | --- | --- | --- | --- | --- |
| **Variable** | **Mean ± S.E. ^a^** | **Range** | **25^th^** | **50^th^ (Median)** | **75^th^** |
| miR194 in primary tumors (N=234) | 2.67 ± 0.90 | 0.002 – 207.38 | 0.37 | 0.86 | 1.92 |
| miR194 in metastatic tumors (N=12) | 1.30 ± 0.37 | 0.35 – 4.91 | 0.40 | 0.97 | 1.47 |
| *P=*0.829^b^ | | | | | |

^a^ Standard error of the mean.

^b^ Calculated using the Mann-Whitney *U* test.

**Supplementary Table 2.**miR-194is predicted to target key molecules and pathways involved in RCC progression.

| **Pathway** | **Candidate Gene Name** |
| --- | --- |
| HIF-1 signaling pathway | *CAMK2G, IGF1R , AKT2,EIF4E* |
| mTOR signaling pathway | *RRAGD, EIF4E, IGF1, RICTOR, MAPK1, RPS6KA3, PDPK1, HIF1A, TSC1, RPS6KA2, RHEB, PRKAA1, CAB39, PIK3R1, AKT3, AKT2* |
| VEGF signaling pathway | *PRKCA, MAPK1, PTGS2, RAC1, NFAT5, PPP3R1, MAPK11, PPP3CA, CHP, AKT3, PIK3R1, PLA2G2F, NFATC1, AKT2* |
| TGF-beta signaling pathway | *LTBP1, TGFBR1, GDF6, SMAD5, CREBBP, BMPR2, SMAD3, RBX1, MAPK1, ACVR2B, ZFYVE9, ID4, SMURF1, THBS1, BMPR1B, THBS2, BMPR1A, ACVR1* |
| Apoptosis | *XIAP, DFFA, PPP3R1, FASLG, FADD, ATM, CASP7, BCL2, RIPK1, PRKAR1A, PPP3CA, CHP, PIK3R1, AKT3, AKT2* |
| Wnt signaling pathway | *WNT5A, BTRC, CAMK2G, PPP3R1, TCF7L2, RBX1, CHD8, CSNK2A1, RAC1, NFAT5, PPP3CA, CHP, SOX17, NFATC1, APC, DVL2, PRKCA, TBL1XR1, VANGL1, CREBBP, CSNK1A1L, SMAD3, FZD5, FZD4, FZD6, SENP2, CTNNBIP1, CCND1, CCND2, SFRP2, PRICKLE2, LRP6* |
| Bladder cancer | *FGFR3, THBS1, E2F3* |
| Melanoma | *E2F2, FGF5, FGFR1, E2F3, PDGFA, MITF, IGF1, CDK6, FGF12, PTEN, IGF1R, MAPK1, CCND1, PDGFRA, MDM2, FGF2, PIK3R1, AKT3, AKT2* |
| Pancreatic cancer | *AKT2, E2F3, RAC1* |
| Basal cell carcinoma | *HHIP, FZD6, SUFU, SHH* |
| Jak-STAT signaling pathway | *SOCS2, SPRED1, STAT5B, AKT2 , SOCS5, IL6ST, IL10* |
| NF-kappa B signaling pathway | *TNFSF11, TRAF6* |
| Toll-like receptor signaling pathway | *AKT2, TRAF6, RAC1* |
| Prostate cancer | *E2F2, FGFR1, E2F3, AR, KLK3, PDGFA, CREBBP, FOXO1, IGF1, TCF7L2, PTEN, CCNE1, MAPK1, IGF1R, PDPK1, CCND1, CDKN1B, BCL2, PDGFRA, NKX3-1, MDM2, PIK3R1, AKT3, AKT2* |
| Small cell lung cancer | *TRAF1, E2F2, CKS1B, E2F3, XIAP, PTGS2, RXRA, CDK6, ITGB1, PTEN, CCNE1, CCND1, CDKN1B, ITGA6, LAMC3, BCL2, LAMC1, TRAF6, PIK3R1, TRAF4, AKT3, AKT2* |
| Non-small cell lung cancer | *PRKCA, E2F2, MAPK1, PDPK1, E2F3, CCND1, RXRA, CDK6, FOXO3, STK4, AKT3, PIK3R1, AKT2* |
| Focal adhesion | *PIK3R2, XIAP, TLN2, PDGFA, DIAPH1, PIP5K1C, ITGB1, PTEN, CHAD, ACTG1, IGF1R, PDPK1, ARHGAP5, PAK2, ITGB8, BCL2, RAC1, COL6A3, THBS1, COL11A1, THBS2, AKT3, PIK3R1, AKT2, PRKCA, ACTB, IGF1, PPP1CB, MAPK1, ITGA9, CCND1, ITGA6, CCND2, LAMC3, PDGFRA, RAP1B, LAMC1, CRK, PARVA* |
| Colorectal cancer | *DVL2, TGFBR1, SMAD3, BIRC5, FZD5, APPL1, FZD4, TCF7L2, FZD6, MAPK1, IGF1R, CCND1, BCL2, RAC1, PDGFRA, PIK3R1, AKT3, APC, AKT2* |
| MAPK signaling pathway | *MEF2C, FGFR1, FGF5, FGFR3, PDGFA, DUSP10, PPP3R1, CACNB1, CACNB2, FASLG, FGF12, SRF, PAK2, MAP3K2, RASGRP1, RAC1, PPP3CA, CHP, TRAF6, FGF2, AKT3, AKT2, PRKCA, TAOK1, TGFBR1, RELB, NF1, MAPK11, STK4, MAPK1, MAP4K4, RPS6KA3, DUSP1, RPS6KA2, NTRK2, PDGFRA, CACNA1E, RAP1B, STMN1, CACNA1C, DUSP9, CRK, PLA2G2F* |
| ECM-receptor interaction | *SDC4, ITGB1, CHAD, HMMR, CD47, ITGA9, ITGA6, ITGB8, LAMC3, COL6A3, GP1BA, LAMC1, THBS1, THBS2, COL11A1* |
| Pancreatic cancer | *E2F2, E2F3, TGFBR1, ARHGEF6, SMAD3, CDK6, RAD51, MAPK1, CCND1, RAC1, PIK3R1, AKT3, AKT2* |
